# Revised Definitions of Tuberculosis Resistance and Treatment Outcomes, France, 2006–2019

**DOI:** 10.3201/eid2809.220458

**Published:** 2022-09

**Authors:** Yousra Kherabi, Mathilde Fréchet-Jachym, Christophe Rioux, Yazdan Yazdanpanah, Frédéric Méchaï, Valérie Pourcher, Jérôme Robert, Lorenzo Guglielmetti, for the MDR-TB Management Group

**Affiliations:** Assistance Publique Hôpitaux de Paris, Hôpital Bichat-Claude Bernard, Paris, France (Y. Kherabi, C. Rioux, Y. Yazdanpanah);; Sorbonne Université, INSERM U1135, Paris (Y. Kherabi, J. Robert, L. Guglielmetti);; Centre Hospitalier de Bligny, Briis-sous-Forges, France (M. Frechet-Jachym);; Assistance Publique—Hopitaux de Paris, Paris (C. Rioux, Y. Yazdanpanah);; INSERM U1137, IAME, Université de Paris, Paris (Y. Yazdanpanah);; Universite Paris Diderot, Paris (Y. Yazdanpanah);; Assistance Publique Hôpitaux de Paris, Hôpital Avicenne, Paris (F. Méchaï);; Hopital Avicenne, Bobigny, France (F. Méchaï);; Assistance Publique Hôpitaux de Paris, Hôpital Pitié-Salpêtrière, Paris (V. Pourcher);; Assistance Publique Hôpitaux de Paris, Sorbonne-Université, Hôpital Pitié-Salpêtrière, Paris (J. Robert, L. Guglielmetti)

**Keywords:** tuberculosis, MDR TB, XDR TB, bedaquiline, linezolid, tuberculosis and other mycobacteria, respiratory infections, antimicrobial resistance, France, bacteria

## Abstract

Definitions of resistance in multidrug-resistant tuberculosis (MDR TB) and extensively drug-resistant tuberculosis (XDR TB) have been updated. Pre–XDR TB, defined as MDR TB with additional resistance to fluoroquinolones, and XDR TB, with additional resistance to bedaquiline or linezolid, are frequently associated with treatment failure and toxicity. We retrospectively determined the effects of pre-XDR/XDR TB resistance on outcomes and safety of MDR TB treatment in France. The study included 298 patients treated for MDR TB at 3 reference centers during 2006–2019. Of those, 205 (68.8%) cases were fluoroquinolone-susceptible MDR TB and 93 (31.2%) were pre-XDR/XDR TB. Compared with fluoroquinolone-susceptible MDR TB, pre-XDR/XDR TB was associated with more cavitary lung lesions and bilateral disease and required longer treatment. Overall, 202 patients (67.8%) had favorable treatment outcomes, with no significant difference between pre-XDR/XDR TB (67.7%) and fluoroquinolone-susceptible MDR TB (67.8%; p = 0.99). Pre-XDR/XDR TB was not associated with higher risk for serious adverse events.

Rifampin-resistant tuberculosis (RR TB) and multidrug-resistant tuberculosis (MDR TB), defined as TB resistant to both rifampin and isoniazid, are a global public health threat. In 2019, there were 465,000 incident cases of RR TB, among which 78% were MDR TB (*1*). In France, the yearly incidence of MDR TB cases was stable at ≈50 cases during 2006–2010, dramatically increased in the next 4 years (*2*) to >100 cases in 2014 (*3*), and slightly decreased afterwards to 75 cases in 2019 (*4*).

RR/MDR TB cases are difficult to treat, and patients need prolonged treatment courses, which are burdened by frequent drug-related adverse events. Global treatment success for RR/MDR TB was 59% in 2018 (*1*). At that time, fluoroquinolones were considered the cornerstone of RR/MDR TB treatment. In 2018, the World Health Organization (WHO) published new treatment guidelines, relying on a large-scale meta-analysis (*5*), which revolutionized the traditional hierarchy of anti-TB drugs (*6*). In those guidelines, newer and repurposed drugs, such as bedaquiline and linezolid, were recommended for all MDR TB patients in addition to fluoroquinolones; second-line injectables would be reserved for cases where no other options are available. 

Globally, 16.2% of RR/MDR TB isolates have acquired resistance to fluoroquinolones (*1*), indicating the need for an update in resistance definitions. Thus, in January 2021, WHO defined pre–extensively drug-resistant TB (pre-XDR TB) as MDR TB with additional resistance to fluoroquinolones, and XDR TB as pre-XDR TB with additional resistance to >1 additional group A drug (bedaquiline and linezolid as of July 2022) (*7*).

Resistance to fluoroquinolones is classically considered a risk factor for treatment failure (*5*,*8*,*9*). However, recent studies from countries with both low (*10*) and high (*11*) TB prevalence did not confirm this finding in high-income settings in which diagnostics and group A drugs are widely available. Furthermore, evidence on the effect of fluoroquinolone resistance on treatment safety is scarce (*11*).

We assessed whether pre-XDR TB and XDR TB status (i.e., additional resistance to any fluoroquinolone) affected outcomes and safety of MDR TB treatment for TB in France, a high-income, low TB incidence country. The Institutional Review Board of the Bligny Hospital (Briis-sous-Forges, France) granted ethical clearance (CRE 2021 08).

## Methods

### Study Design and Participants

In this retrospective observational cohort study, we included all consecutive MDR TB patients confirmed at the French National Reference Center for Mycobacteria (NRC; Paris, France) who initiated treatment at 3 referral hospitals (Bligny, in Briis-sous-Forges, and Pitié-Salpêtrière and Bichat-Claude Bernard, both in Paris) during January 1, 2006–December 31, 2019. Patients were followed up for 2 years after the end of treatment. We considered only the first episode of MDR TB within the study period. We retrieved the latest data on December 31, 2021.

We reviewed medical records to retrieve demographic and clinical features, as well as results of laboratory, radiographic, and microbiological tests. We extracted routinely collected data from medical records and anonymized the data; the investigator who extracted data was not involved in patient care. We obtained comprehensive drug susceptibility testing (DST) profiles from the database of the NRC laboratory.

### Definitions 

We performed phenotypic DST at the NRC using the Löwenstein–Jensen proportion method (*12*). We obtained genotypic DST through commercially available line-probe assays (GenoType MTBDRplus and GenoType MTBDRs; Hain Lifescience, https://www.hain-lifescience.de) or targeted DNA sequencing. We used new definitions of drug-resistant TB (*7*): we defined MDR TB as TB resistant to isoniazid and rifampin, pre-XDR TB as MDR TB with resistance to any fluoroquinolone, and XDR TB as MDR TB with additional resistance to any fluoroquinolone and another WHO group A drug (bedaquiline or linezolid). We classified patients as new or previously treated according to WHO definitions. Sputum smear and cultures were routinely performed at treatment start, every 14 days until culture conversion, and monthly thereafter. We defined sputum culture conversion as 2 consecutive negative cultures collected at least 30 days apart. We defined treatment outcomes using 2020 WHO definitions (*13*). Of note, we defined treatment outcome as not evaluated for patients for whom no treatment outcome was assigned; this definition includes cases transferred out to another treatment unit and those whose treatment outcome was unknown but excludes those who did not attend follow-up appointments and did not respond to attempted contact from clinical staff, which we categorized as lost to follow-up.

### Treatment Regimens

Throughout the study period, treatment regimens were designed in accordance with WHO treatment guidelines and in consultation with the French MDR TB Consilium, a multidisciplinary team coordinated by the France NRC (*14*–*17*). Of note, linezolid has been used for MDR TB treatment in France since 2006 and bedaquiline since 2011. Drugs were considered to be part of the treatment regimen if they were administered for >30 days.

### Outcomes

The primary study outcome was the proportion of treatment success (defined as the sum of cured and treatment completed). Secondary outcomes were time to sputum culture conversion and proportion of treatment-emergent serious adverse events (SAE), as defined by the US Food and Drug Administration (*18*).

### Statistical Analysis

#### Sample Size

Based on NRC surveillance data for the period under review, we estimated that ≈300 MDR TB patients, 200 with fluoroquinolone-susceptible MDR TB and 100 with pre-XDR/XDR TB, could be included. Taking into account the results of a large meta-analysis comparing MDR TB treatment outcomes according to different resistance patterns (*5*), we estimated a relative risk of 1.59 for an unfavorable treatment outcome in pre-XDR/XDR TB compared with fluoroquinolone-susceptible MDR TB cases. With these assumptions, the expected sample size would provide a power of 93% with a 2-sided α risk of 2.5% to detect a difference between the 2 groups.

#### Statistical Methods

We collected the extracted data on standardized forms, entered them into a database located at the NRC, and ran analyses using Stata version 16 (StataCorp, https://www.stata.com). We performed sensitivity analyses focusing on patients treated after 2011, the year bedaquiline was introduced in France.

For descriptive statistics, we reported continuous variables as medians with interquartile range (IQR) and compared them using the Wilcoxon rank-sum test or 2-sample t-test, as appropriate. We reported categorical variables as frequencies with percentages and compared using the Fisher exact test or χ^2^ test, as appropriate. A 2-sided α<0.05 was considered statistically significant. Because the cohort included few XDR TB patients, we performed most analyses by grouping together pre-XDR TB and XDR TB cases.

To identify risk factors associated to the dependent categorical variables (treatment success and SAE), we fitted unconditional logistic regression models. We included explanatory variables in the initial models if associated with the dependent variable at p<0.20 in univariate analysis. We then performed backward analysis: we kept explanatory variables associated with the dependent variable that had p<0.20, in addition to the variable of interest (pre-XDR/XDR TB), in the model. 

As secondary objectives, we performed survival analyses to describe time from treatment start to sputum culture conversion (for patients with positive sputum cultures at treatment start) and unfavorable treatment outcome (for all patients). We estimated Kaplan-Meier curves and performed log-rank tests to assess the effects of pre-XDR/XDR TB. We fitted multivariable Cox proportional-hazard models to identify predictive factors of sputum culture conversion. We included variables into multivariable Cox proportional hazards models if they predicted the outcome at p<0.20 in univariate analysis and if they fulfilled the proportional hazards assumption at baseline or, if appropriate, after addition of a time interaction. We reported hazard ratios with 95% CI.

For both logistic regression and Cox models, we performed complete case analyses to identify the variables included in the model and then imputed missing data with multiple imputation using chained equations with 10 imputed datasets. Overall, the proportion of missing observations in the included variables was 0%–9%.

## Results

### Population Characteristics

The study population was made up of 298 MDR TB patients, including 84 (28.2%) with pre-XDR TB and 9 (3.0%) with XDR TB. Sixty-six patients (22.1%) were treated during 2006–2010 and 232 (77.9%) during 2011–2019. The median age at admission was 34 years (IQR 27–42), and 202 (67.8%) patients were male. Patients were mainly born in the WHO Europe region (54.6%) (Table 1). Pre-XDR/XDR TB patients were more frequently born in the WHO Europe region; had more frequently precarious housing, past imprisonment history, and active drug abuse (alcohol, smoking, intravenous drugs); and had more severe pulmonary tuberculosis, including cavitary lesions and bilateral disease. Patients with pre-XDR/XDR TB more frequently had additional resistance to cycloserine, second-line injectables, pyrazinamide, and P-aminosalicylic acid.

**Table 1:** Characteristics and demographics of patients affected by MDR TB, France, 2006–2019*

Characteristic	Total, N = 298	TB resistance status	p value

MDR, n = 205	Pre-XDR/XDR, n = 93
Median age, y (IQR)	34 (27–42)	33 (27–42)	36 (28–42)	NS
Sex				
M	202 (67.8)	130 (63.4)	72 (77.4)	0.02
F	96 (32.2)	75 (36.6)	(22.6)	
WHO Region place of birth				<0.001
Europe	162 (54.6)	85 (41.7)	77 (82.8)	
Africa	95 (32.0)	85 (41.7)	10 (10.8)	
South-East Asia	23 (7.7)	20 (9.8)	3 (3.2)	
Others	18 (6.0)	15 (7.3)	3 (3.2)	
Precarious housing or homeless	177 (59.6)	105 (51.5)	72 (77.4)	<0.001
Past imprisonment	22 (7.4)	7 (3.3)	15 (16.1)	<0.001
Comorbidities				
HIV infection	29 (9.8)	20 (9.8)	9 (9.8)	NS
HBV infection	21 (7.1)	13 (6.4)	8 (8.7)	NS
HCV infection	46 (15.5)	17 (8.3)	29 (31.2)	<0.001
Immunosuppressive condition	14 (4.8)	8 (4.0)	6 (6.5)	NS
Immunosuppressive treatment	8 (2.7)	6 (2.9)	2 (2.1)	NS
Diabetes	13 (4.4)	9 (4.4)	4 (4.3)	NS
Psychiatric disorder	23 (7.7)	10 (4.9)	13 (14.0)	<0.001
Albumin, median, g/L (IQR)	34 (31–38)	35 (31–39)	33 (30–37)	NS
BMI, median, kg/m^2^ (IQR)	20.1 (18.4–22.2)	20.2 (18.6–22.3)	19.8 (17.9–21.9)	NS
Previous TB treatment	159 (53.5)	90 (44.1)	69 (74.2)	<0.001
Previous TB treatment with second-line drugs	89 (45.2)	35 (28.9)	54 (71.1)	<0.001
Other risk factors				
Alcohol abuse	37 (12.5)	20 (9.8)	17 (18.3)	0.002
Active smoking	103 (34.7)	54 (26.5)	49 (52.7)	<0.001
IVDU	44 (14.8)	17 (8.3)	27 (29.0)	<0.001

*Values are presented as no. (%), unless indicated otherwise. BMI, body-mass index; IQR, interquartile range; IVDU, intravenous drug use; MDR, multidrug resistant (susceptible to all fluoroquinolones); NS, not statistically significant; pre-XDR/XDR = pre–extensively drug resistant/extensively drug resistant, (resistant to >1 fluoroquinolone); TB, tuberculosis; WHO, World Health Organization.

### Treatment and Outcomes

Overall, 94.3% patients were treated with >1 group A drug (levofloxacin, moxifloxacin, bedaquiline, or linezolid) and 76.8% with >1 group B drug (clofazimine or cycloserine). Linezolid (89.3% vs. 69.8%, p<0.001) and bedaquiline (74.2% vs. 28.3%, p<0.001) were more frequently used in pre-XDR/XDR TB cases. A large part of our study population received second-line injectables (85.2%), including similar proportions in the 2 groups of interest (81.7% vs. 86.8%). Total treatment duration was longer in pre-XDR/XDR TB patients (median 21.2 months vs. 17.3 months; p<0.001) (Table 2).

**Table 2:** Baseline disease characteristics for 298 patients affected by MDR TB, France, 2006–2019*

Characteristic	Total, N = 298	TB resistance status	p value

MDR, n = 205	Pre-XDR/XDR, n = 93
TB characteristics				
Pulmonary tuberculosis	272 (91.6)	179 (87.8)	93 (100)	0.002
Bilateral lung involvement, n = 233	174 (74.7)	100 (71.0)	74 (86.0)	0.009
Cavitary lung disease, n = 224	156 (69.6)	82 (59.4)	74 (86.0)	<0.001
Miliary TB	11 (3.8)	8 (4.0)	3 (3.2)	NS
Sputum smear positive†	202 (68.0)	118 (57.8)	84 (90.3)	<0.001
Sputum culture positive†	214 (71.8)	132 (64.4)	82 (88.2)	<0.001
Extrapulmonary TB	83 (28.1)	69 (34.0)	14 (15.2)	0.003
Additional drug resistance				
Any second-line injectable, n = 287‡	83 (28.9)	32 (16.3)	51 (56.0)	<0.001
Linezolid, n = 260	5 (1.9)	1 (0.6)	4 (4.5)	NS
Bedaquiline, n = 101	6 (5.9)	0	6 (19.4)	<0.001
Linezolid and/or bedaquiline, n = 260	10 (3.8)	1 (0.6)	9 (10.1)	<0.001
Pyrazinamide, n = 183	109 (59.6)	65 (49.6)	44 (84.6)	<0.001
Ethambutol, n = 287	177 (61.7)	104 (53.1)	73 (80.2)	<0.001
Ethionamide or prothionamide, n = 276	175 (63.4)	113 (60.1)	62 (70.5)	NS
Cycloserine, n = 279	70 (25.1)	26 (13.6)	44 (50.0)	<0.001
P-aminosalicylic acid, n = 262	33 (20.4)	17 (9.6)	16 (18.8)	0.04
Delamanid, n = 26	2 (7.7)	1 (5.3)	1 (14.3)	NS
Anti-TB treatment				
Treatment regimens				
Fluoroquinolones	218 (73.2)	182 (88.8)	36 (38.7)	<0.001
Levofloxacin	56 (18.8)	43 (21.0)	13 (14.0)	NS
Moxifloxacin 400 mg/day	187 (62.8)	167 (81.5)	20 (21.5)	<0.001
Moxifloxacin 800 mg/day	14 (4.7)	3 (1.4)	11 (11.8)	<0.001
Linezolid	226 (75.8)	143 (69.8)	83 (89.3)	<0.001
Bedaquiline	127 (42.6)	58 (28.3)	69 (74.2)	<0.001
Total treatment duration, mo, median (IQR), n = 228	17.8 (13.4–20.6)	17.3 (13.1–18.1)	21.2 (16.7–24.0)	<0.001
Duration of treatment with second-line injectable, median mo (IQR), n = 220	4.7 (3.0–7.1)	3.9 (3.0–5.5)	7.7 (4.7–14.0)	NS
Lung surgery	27 (9.9)	8 (4.4)	19 (20.9)	<0.001
Good treatment adherence§	232 (78.4)	161 (79.3)	71 (76.3)	NS

*Values are no. (%) except as indicated. IQR, interquartile range; MDR, multidrug resistant (susceptible to all fluoroquinolones); NS, not statistically significant; pre-XDR/XDR, pre–extensively drug resistant/extensively drug resistant, (resistant to >1 fluoroquinolone); TB, tuberculosis; WHO, World Health Organization. †At treatment start. ‡Amikacin, capreomycin, or kanamycin. §Assessed by treating physician.

Overall, 202 patients (67.8%) had treatment success, including cure (62.1%) and treatment completion (5.7%) (Table 3). Ninety-six patients (32.2%) had unfavorable or other outcomes. Of those, 3 patients had treatment failure and 5 died; 54 were lost to follow-up, and 34 did not have treatment outcomes evaluated (Table 3). Of note, 67.9% of patients with pre-XDR TB and 66.7% patients with XDR TB had treatment success. For the 5 patients who died during TB treatment, causes of death were malignancy progression (2 patients), septic shock (urinary tract sepsis for 1 patient and disseminated candidemia for 1 patient), and decompensated cirrhosis (1 patient). Among patients with treatment success, 145 (71.8%) were followed up to 12 months after end of treatment and 129 (63.9%) up to 24 months. Two patients with pre-XDR/XDR TB relapsed in the first year and 1 in the second year of follow-up after end of treatment. One patient with fluoroquinolone-susceptible MDR TB relapsed in the second year of follow-up.

**Table 3:** Tuberculosis treatment outcomes of patients affected by MDR TB, France, 2006–2019*

Characteristic	Total, N = 298	TB resistance status	p value

MDR, n = 205	Pre-XDR/XDR, n = 93
Treatment success	202 (67.8)	139 (67.8)	63 (67.7)	NS
Cure	185 (62.1)	126 (61.5)	59 (63.4)	NS
Treatment completed	17 (5.7)	13 (6.3)	4 (4.3)	NS
Sustained treatment success at 12 mo†, n = 145	143 (98.6)	101 (100)	42 (95.5)	NS
Treatment failure	3 (1.0)	1 (0.5)	2 (2.2)	NS
Others	93 (31.2)	65 (31.7)	28 (30.1)	NS
Died	5 (1.7)	2 (1.0)	3 (3.2)	NS
Lost to follow-up‡	54 (18.1)	36 (17.6)	18 (19.4)	NS
Not evaluated	34 (11.4)	27(13.2)	7 (7.5)	NS
Days to sputum culture conversion, n = 198§ (IQR)	60 (38–90)	50 (31–73)	88 (51–108)	<0.001

*Values are no (%) except as indicated. IQR, interquartile range; MDR, multidrug resistant (susceptible to all fluoroquinolones); NS, not statistically significant; pre-XDR/XDR = pre–extensively drug resistant/extensively drug resistant, (resistant to >1 fluoroquinolone); TB, tuberculosis. †Among patients with treatment success at end of treatment. ‡These patients did not attend follow-up appointments and did not respond to attempted contact from clinical staff. §Among patients with a positive sputum culture at treatment start.

### Predictors of Treatment Outcome

Multivariable analysis showed that pre-XDR/XDR TB status was not independently associated with treatment outcome (adjusted odds ratio [aOR] 0.81, 95% CI 0.47–1.41); conversely, previous anti-TB treatment (aOR 1.80, 95% CI 1.13–2.89) and poor treatment adherence (aOR 1.19, 95% CI 1.07–1.33) were independently associated with unfavorable treatment outcome (Table 4). Multivariable Cox proportional hazard model for unfavorable outcome was consistent with these findings (Appendix Table 2). We confirmed these results by a sensitivity analysis restricting the sample to patients who started treatment during 2011–2019 (Appendix Table 3).

**Table 4:** Risk factors for unsuccessful outcomes in patients affected by MDR TB, France, 2006–2019*

Characteristic	aOR (95% CI)	p value
TB resistance status		
Fluoroquinolone-susceptible MDR	Referent	
Pre-XDR/XDR	0.81 (0.47–1.41)	0.48
History of anti-TB treatment		
No	Referent	
Yes	2.16 (1.32–3.55)	0.002
Treatment adherence†		
Good	Referent	
Poor	1.21 (1.08–1.35)	0.001
HCV coinfection		
No	Referent	
Yes	0.63 (0.36–1.10)	0.11
Immunosuppression‡		
No	Referent	
Yes	1.56 (0.50–4.89)	0.44
Pulmonary tuberculosis		
No	Referent	
Yes	2.92 (0.90–9.50)	0.07

*We used multivariable logistic regression with multiple imputation for missing data. Results are from the final model. aOR, adjusted odds ratio; MDR, multidrug resistant (susceptible to all fluoroquinolones); NS, not statistically significant; pre-XDR/XDR, pre–extensively drug resistant/extensively drug resistant, (resistant to >1 fluoroquinolone); TB, tuberculosis. †Assessed by treating physician. ‡Immunosuppressive disease and/or immunosuppressive treatment.

### Sputum Culture Conversion

Among 214 patients with positive sputum culture at baseline, pre-XDR/XDR TB patients showed longer time to sputum culture conversion than MDR TB patients (median 88 days vs. 50 days; p = 0.001 by log rank test) (Figure). Time to sputum culture conversion in XDR TB patients was 114 days (95% CI 60–191 days). In a multivariable Cox proportional hazard model, pre-XDR/XDR TB (adjusted hazard ratio [aHR] 0.59, 95% CI 0.42–0.84) and alcohol abuse (aHR 0.61, 95% CI 0.38–0.99) were independently associated with slower sputum culture conversion (Table 5).

**Figure:**
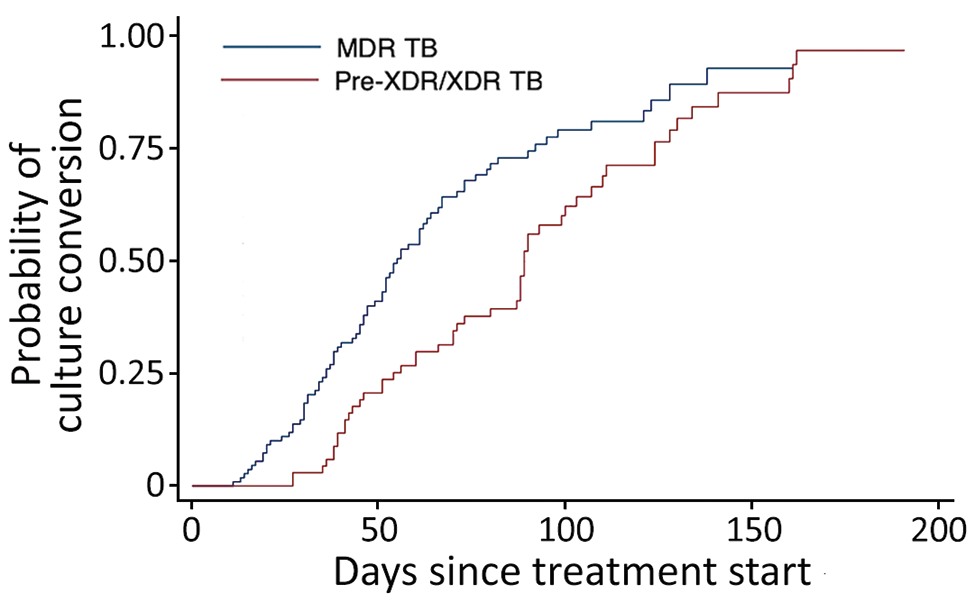
Kaplan–Meier curves of sputum time to culture conversion in fluoroquinolone-susceptible MDR TB and pre-XDR/XDR TB patients (log rank test p = 0.001). MDR, multidrug resistant (susceptible to all fluoroquinolones); pre-XDR/XDR, pre–extensively drug resistant/extensively drug resistant (resistant to >1 fluoroquinolone); TB, tuberculosis.

**Table 5:** Multivariable Cox proportional hazard model for sputum conversion in 214 patients affected by MDR TB and with positive sputum culture at baseline, France, 2006–2019*

Characteristic	aHR (95% CI)	p value
TB resistance status		
Fluoroquinolone-susceptible MDR	Referent	
Pre-XDR/XDR	0.59 (0.42–0.84)	0.003
Diabetes		
No	Referent	
Yes	1.54 (0.79–2.97)	0.20
Treatment adherence†		
Good	Referent	
Poor	1.40 (0.88–2.25)	0.16
Addiction to alcohol		
No	Referent	
Yes	0.61 (0.38–0.99)	0.04

*We used multiple imputation for missing data. Results are from the final model. aHR, adjusted hazard ratio. †Assessed by treating physician.

### Adverse Drug Reactions

Overall, 152 patients (51.0%) had >1 SAE while being treated for MDR TB (Table 6). SAE were more frequent in pre-XDR/XDR TB case-patients than in fluoroquinolone-susceptible MDR TB case-patients (62.4% vs. 45.9%; p = 0.02). Peripheral neuropathy was the most frequent SAE (27.5%), regardless of TB resistance status; in all cases, peripheral neuropathy was attributed to linezolid. Severe ototoxicity occurred more frequently in pre-XDR/XDR TB than in fluoroquinolone-susceptible MDR TB cases (38.7% vs. 20.5%; p = 0.01) (Appendix Table 4); all cases of severe ototoxicity were attributed to amikacin. In multivariable analysis (Table 7), pre-XDR/XDR TB was not independently associated with severe toxicity (aOR 1.31, 95% CI 0.76–2.26; p = 0.34), whereas poor treatment adherence was associated with a higher risk for SAE (aOR 1.24 95% CI 1.04–1.47; p = 0.01).

**Table 6:** Serious adverse events of 298 patients affected by MDR TB, France, 2006–2019*

Characteristic	Total, N = 298	TB resistance status		p value
		MDR, n = 205	Pre-XDR/XDR, n = 93	
Serious adverse events	152 (51.0)	94 (45.9)	58 (62.4)	0.02
Event type				
Peripheral neuropathy	82 (27.5)	50 (24.4)	32 (34.4)	NS
Ototoxicity	78 (26.2)	42 (20.5)	36 (38.7)	0.001
Gastrointestinal	39 (13.1)	26 (12.7)	16 (17.2)	NS
Hepatotoxicity	35 (11.7)	27 (13.2)	8 (8.6)	NS
Hematologic abnormalities	29 (9.7)	17 (8.3)	12 (13.0)	NS
Anemia	20 (6.7)	12 (5.9)	8 (8.6)	NS
Thrombocytopenia	11 (3.7)	5 (2.4)	6 (6.5)	NS
Neutropenia	6 (2.0)	4 (2.0)	2 (2.2)	NS
Musculoskeletal pain	24 (8.1)	20 (9.8)	4 (4.3)	NS
Tendinopathy	10 (3.4)	9 (4.4)	1 (1.1)	NS
Arthralgia	15 (5.0)	12 (5.9)	3 (3.2)	NS
Psychiatric	18 (6.0)	12 (5.9)	6 (6.5)	NS
Renal toxicity	17 (5.7)	14 (6.8)	3 (3.2)	NS
Optic neuritis	10 (3.4)	5 (2.4)	5 (5.4)	NS
Hypothyroidism	9 (3.0)	6 (2.9)	3 (3.2)	NS
QT prolongation	8 (2.7)	4 (2.0)	4 (4.3)	NS
Other	14 (4.7)	4 (2.0)	10 (10.8)	NS

*Values are no (%) except as indicated. MDR, multidrug resistant (susceptible to all fluoroquinolones); NS, not statistically significant; QT, QT interval on electrocardiogram; pre-XDR/XDR, pre–extensively drug resistant/extensively drug resistant, (resistant to >1 fluoroquinolone); TB, tuberculosis.

**Table 7:** Risk factors for serious adverse events in 298 patients affected by MDR TB, France, 2006–2019*

Characteristic	aOR (95% CI)	p value
TB resistance status		
Fluroroquinolone-susceptible MDR	Referent	
Pre-XDR/XDR	1.31 (0.76–2.26)	0.34
Treatment adherence†		
Good	Referent	
Poor	1.24 (1.04–1.47)	0.01
Past imprisonment		
No	Referent	
Yes	1.15 (0.92–1.44)	0.21
Pulmonary tuberculosis		
No	Referent	
Yes	0.71 (0.47–1.09)	0.18

*We used multivariable logistic regression with multiple imputation for missing data. Results are from the final model. aOR, adjusted odds ratio; MDR, multidrug resistant (susceptible to all fluoroquinolones); pre-XDR/XDR, pre–extensively drug resistant/extensively drug resistant, (resistant to >1 fluoroquinolone); TB, tuberculosis. †Assessed by treating physician.

## Discussion

Overall, we did not detect a significant effect of pre-XDR and XDR TB resistance, in accordance with the revised WHO definitions, on treatment outcomes of MDR TB in France during 2006–2019. The global treatment success proportion in MDR TB patients was 67.8%; previous TB treatment and poor treatment adherence were each independently associated with unfavorable outcome. Pre-XDR/XDR TB patients showed longer time to sputum culture conversion compared with fluoroquinolone-susceptible MDR TB. Pre-XDR/XDR TB resistance status was not independently associated with higher risk for SAEs.

In the literature, pre-XDR/XDR TB has been consistently associated with treatment failure (*19*–*22*). Indeed, in a meta-analysis published by Ahmad et al., the pooled successful treatment proportion for fluoroquinolone-susceptible MDR TB was estimated at 62%–73%, whereas that of pre-XDR/XDR TB was 51%–57% during the same period (2009–2016) (*5*). However, MDR/XDR TB treatment outcomes have improved with the introduction of bedaquiline and delamanid (*23*–*25*), together with repurposed TB drugs such as linezolid, clofazimine, and carbapenems (*26*–*29*). In our cohort, treatment success proportions were comparable between fluoroquinolone-susceptible MDR TB, pre-XDR TB, and XDR TB patients. These results are consistent with previous studies from high-income countries such as Italy (*10*) and South Korea (*11*). In those countries, as in France, these results may be explained by the routine availability of rapid molecular testing, therapeutic drug monitoring, newer and repurposed drugs, and treatment design in consultation with a MDR TB consilium (*16*,*17*,*30*).

The finding that fluoroquinolone resistance does not affect treatment outcomes reflects the early access and use of new and repurposed drugs in France. However, the proportion of treatment success in our cohort (67.8%) may appear low compared with clinical trials, which usually achieve >80% success (*31*,*32*). A likely explanation is the high proportion of lost-to-follow-up (18.1%) and nonevaluated (11.4%) outcomes, which account for 92% of all unfavorable outcomes in our real-world cohort. This reasoning is consistent with previous findings showing that even high-income settings have room to improve with respect to supports provided to MDR TB patients (*33*), especially when a high proportion is not locally born and may not be permanent residents. 

In our study, pre-XDR/XDR TB was associated with substantially longer time to sputum culture conversion than was fluoroquinolone-susceptible MDR TB. However, the difference did not affect treatment outcomes; this finding is consistent with previous studies in which time to culture conversion was not a good predictor of treatment outcome (*34*,*35*). In our study, the pre-XDR/XDR TB and fluoroquinolone-susceptible MDR TB populations were very different. Indeed, pre-XDR/XDR TB patients had more severe TB pulmonary disease, as previously reported (*10*,*11*). In addition, social indicators of precarity and psychiatric disorders were more frequently observed in the pre-XDR/XDR TB population. Despite these differences in baseline characteristics that we expected to cause worse outcome in pre-XDR/XDR TB patients, fluoroquinolone-resistant MDR TB patients yielded treatment success rate similar to that of fluoroquinolone-susceptible MDR TB patients, and pre-XDR/XDR TB resistance was not independently associated with unfavorable treatment outcomes.

Our study also highlights that the revised definition of XDR TB concerns a limited number of patients in France, as previously reported (*36*). Indeed, only 9 patients >14 years of age, corresponding to 3% of the total MDR TB patients, could be classified as XDR TB in our cohort. The small number of patients affected by XDR TB prevented us from performing planned subgroup analyses. Overall, treatment outcome and safety in this group seemed comparable with other MDR TB patients, whereas time to sputum culture conversion was longer.

In multivariable logistic regression, previous TB treatment and poor treatment adherence, but not pre-XDR/XDR TB status, were independently associated with treatment failure. In the literature, numerous risk factors were reported for treatment failure, including age, lower body mass index, history of drug abuse, and comorbidities (*10*,*11*,*37*,*38*). We tested and confirmed the absence of interaction between previous TB treatment and poor treatment adherence before including those variables in multivariable models; however, we could hypothesize that previous treatment that led to poor outcome could be associated with lower adherence to medical visits (i.e., a patient’s lack of confidence in treatment after failure of a previous TB treatment).

A strength of our study is that it includes a multivariable evaluation of safety of treatment in fluoroquinolone-susceptible MDR TB compared with pre-XDR/XDR TB (*11*). In univariate analysis, serious adverse events were more frequent in pre-XDR/XDR TB cases than in fluoroquinolone-susceptible MDR TB cases, but this difference was not confirmed in multivariable analysis. The higher proportion of SAE in the pre-XDR/XDR group is likely linked to a longer duration of treatment and to a more frequent use of poorly tolerated drugs, such as linezolid, in this group, as described previously (*39*).

Our analysis had the inherent limitations of retrospective studies; one was the issue of missing data, which we managed using multiple imputation. Overall, the rate of loss to-follow-up was high in our cohort, and we were not able to collect data up to 2 years after end-of-treatment outcome for all patients. Furthermore, we focused on retrieving only SAEs, without considering nonserious adverse events that, for instance, might have compromised treatment adherence. In addition, we included 3 referral centers in the metropolitan Paris area. A large part of the more complex patients from all areas in France are known to be referred to those 3 centers. Hence, we have a potential bias in extrapolating our conclusions to all cases of MDR TB in France.

In summary, in our cohort of MDR TB patients treated in France during 2006–2019, the proportion of treatment success was only 67.8% because of high rates of patients who did not complete follow-up and unevaluated outcomes. We were unable to detect any differences in success rates between fluoroquinolone-susceptible MDR TB and pre-XDR/XDR TB patients; this finding may be linked to the high proportion of pre-XDR/XDR TB patients receiving newly defined group A drugs and to individually tailored treatment regimens through a multidisciplinary consilium. As expected, SAEs were frequent and affected more than half our cohort patients, which underlines the need for better management of the more toxic drugs such as linezolid.

Appendix Additional information about revised definitions for TB drug resistance and treatment outcomes, France, 2006–2019.
