# Supplementary material for: Revised Definitions of Tuberculosis Resistance and Treatment Outcomes, France, 2006–2019
Source: Emerg Infect Dis. 2022 Sep;28(9):1796–804. doi: 10.3201/eid2809.220458 (PMC9423894; doi:10.3201/eid2809.220458)
Supplement: Appendix — Additional information about revised definitions for TB drug resistance and treatment outcomes, France, 2006–2019. [file 22-0458-Techapp-s1.pdf]

Article DOI: https://doi.org/10.3201/eid2809.220458

Revised Definitions of Tuberculosis Resistance and Treatment Outcomes, France, 2006–2019

Appendix

Appendix Table 1. Antituberculosis drugs, by treatment period, in 298 patients affected by multidrug-resistant tuberculosis France, 2006–2019*

	Treatment period†			
Drug	Total, N = 298	2006–2010, N = 66	2011–2019, N = 232	p value
Ethambutol	133 (44.6)	37 (62.1)	96 (41.4)	0.03
Pyrazinamide	163 (54.7)	35 (53.0)	128 (55.2)	NS
Fluoroquinolones	218 (73.2)	49 (74.2)	169 (72.8)	NS
Levofloxacin	56 (18.8)	4 (6.1)	52 (22.4)	0.003
Moxifloxacin 400 mg/day	187 (62.8)	46 (69.7)	141 (60.8)	NS
Moxifloxacin 800 mg/day	14 (4.7)	0	14 (6)	0.04
Second-line injectable	254 (85.2)	64 (97.0)	190 (81.9)	0.001
Amikacin	244 (81.9)	62 (93.9)	182 (78.5)	0.003
Capreomycin	12 (4.0)	3 (4.6)	9 (3.9)	NS
Ethionamide or prothionamide	87 (29.2)	29 (43.9)	58 (25.0)	0.003
Cycloserine	197 (66.1)	28 (42.4)	169 (72.8)	<0.001
P-aminosalicylic acid	163 (54.7)	49 (74.2)	114 (49.1)	<0.001
Linezolid	226 (75.8)	28 (42.4)	198 (85.3)	<0.001
Meropenem or imipenem and amoxicillin/clavulanate	43 (14.4)	1 (1.5)	42 (18.1)	<0.001
Clofazimine	126 (42.3)	1 (1.5)	125 (53.9)	<0.001
Delamanid	18 (6.0)	0	18 (7.8)	0.02
Bedaquiline	127 (42.6)	0	127 (54.3)	<0.001

*Categorical variables are presented as no. (%). NS, not statistically significant.
†At treatment start.

Appendix Table 2. Multivariable Cox proportional hazard model for non-successful outcome in 298 patients affected by multidrug-resistant tuberculosis, France, 2006–2019*

Characteristic	aHR (95% CI)	p value
TB resistance status		
MDR	1 (ref)	NA
Pre-XDR/XDR	1.18 (0.64-2.19)	0.59
History of previous anti-TB treatment		
No	1 (ref)	NA
Yes	2.03 (1.03-3.99)	0.04
Treatment adherence†		
Good	1 (ref)	NA
Poor	3.57 (1.78-6.74)	<0.0001
HIV co-infection		
No	1 (ref)	NA
Yes	0.73 (0.28-1.89)	0.51

*We conducted multivariable Cox regression analyses (multiple imputation for missing data, final model). aHR, adjusted hazard ratio; MDR, multidrug resistant (susceptible to all fluoroquinolones); NS, not statistically significant; pre-XDR/XDR = pre–extensively drug resistant/extensively drug resistant, (resistant to >1 fluoroquinolone); ref, referent; NA, not applicable; TB, tuberculosis.
†Assessed by treating physician.

Appendix Table 3. Risk factors for nonsuccessful outcomes in 232 patients affected by multidrug-resistant tuberculosis, France, 2006–2019*

Characteristic	aOR (95% CI)	p value
TB resistance status		
MDR	1 (ref)	NA
Pre-XDR/XDR	0.68 (0.36-1.30)	0.24
History of previous anti-TB treatment		
No	1 (ref)	NA
Yes	2.40 (1.32-4.34)	0.004
Treatment adherence§		
Good	1 (ref)	NA
Poor	1.18 (1.05-1.34)	0.005
HCV co-infection		
No	1 (ref)	NA
Yes	0.46 (0.23-0.92)	0.03
HIV co-infection		
No	1 (ref)	NA
Yes	1.38 (0.87-2.20)	0.17
Pulmonary tuberculosis		
No	1 (ref)	NA
Yes	3.60 (0.92-13.99)	0.07

*We conducted multivariable logistic regression with multiple imputation for missing data. Results are from the final model. aOR, adjusted odds ratio; MDR, multidrug resistant (susceptible to all fluoroquinolones); NS, not statistically significant; pre-XDR/XDR = pre–extensively drug resistant/extensively drug resistant, (resistant to >1 fluoroquinolone); ref, referent; NA, not applicable; TB, tuberculosis.
†Assessed by treating physician.

Appendix Table 4. Details of drugs causing serious adverse events in 298 patients affected by multidrug-resistant tuberculosis, France, 2006–2019*

		TB resistance status		
Drug	Total, N = 298	MDR, N = 205	Pre-XDR/XDR, N = 93	p value
Ethambutol	7 (2.4)	2 (1.0)	5 (5.4)	0.03
Pyrazinamide	17 (5.7)	15 (7.3)	2 (2.2)	NS
Fluoroquinolones	34 (11.4)	28 (13.7)	6 (6.5)	0.049
Amikacin	26 (8.7)	16 (7.8)	10 (10.8)	NS
Ethionamide or prothionamide	9 (3.0)	5 (2.4)	4 (4.3)	NS
Cycloserine	9 (3.0)	7 (3.4)	2 (2.2)	NS
P-aminosalicylic acid	27 (9.1)	12 (5.9)	15 (16.1)	0.005
Linezolid	98 (32.9)	59 (28.8)	39 (41.9)	0.02
Clofazimine	8 (2.7)	2 (1.0)	6 (6.5)	0.01
Delamanid	1 (0.3)	0	1 (1.1)	NS
Bedaquiline	6 (2.0)	3 (1.5)	3 (3.2)	NS
Others	6 (2.0)	2 (1.0)	4 (4.3)	NS

*Values are no.(%) unless otherwise stated. MDR, multidrug resistant (susceptible to all fluoroquinolones); NS, not statistically significant; pre-XDR/XDR, pre–extensively drug resistant/extensively drug resistant, (resistant to >1 fluoroquinolone); TB, tuberculosis.
